# A 5 kg giant breast lipoma in a 40-year-old woman: a case report

**DOI:** 10.1097/MS9.0000000000001683

**Published:** 2024-01-04

**Authors:** Samit Sharma, Aadesh Rayamajhi, Abhishek Chapagain, Jayan Man Shrestha

**Affiliations:** a Department of Plastic Surgery and Burns, Tribhuvan University Teaching Hospital; b Maharajgunj Medical Campus, Tribhuvan University, Institute of Medicine, Kathmandu, Nepal

**Keywords:** Benign tumour, breast, case report, excision, giant lipoma

## Abstract

**Introduction and importance:**

Lipomas are benign tumours composed of adipocytes surrounded by a thin fibrous capsule. Although they make up 16% of mesenchymal tumours, the occurrence of breast lipomas is uncertain. Giant lipomas, measuring 10 cm or more in diameter or weighing at least 1000 g, commonly occur in the upper back, neck, and thigh, and are rare in the breast. Given its rarity, accurately diagnosing a giant breast lipoma is crucial to prevent potential overtreatment, as it might otherwise be mistaken for a malignant tumour.

**Case presentation:**

A 40-year-old woman presented with a painless, gradually enlarging left breast mass. Physical examination and imaging studies revealed a lipoma-compatible mass. Surgery was performed, and the 5 kg mass was enucleated and identified as a lipoma on histopathology. The patient had an uneventful postoperative recovery and was satisfied with the outcome of the surgery after 2 months of follow-up.

**Clinical discussion:**

Breast lipomas are more common in the 40–60 years age group, with giant lipomas occurring more frequently in the latter half of this age range. They can mimic various breast conditions, including neoplastic and non-neoplastic conditions, and are often treated with surgical excision to avoid recurrence. The location of the lipoma in the breast can be subcutaneous or intramuscular, and preserving the future pedicle of reduction mammoplasty/mastopexy is essential.

**Conclusion:**

Giant breast lipoma is an infrequent condition that can manifests as progressive enlargement of the breast, posing a diagnostic challenge due to its resemblance to various benign or malignant pathologies.

## Introduction

Highlights

- A giant breast lipoma can occur in younger women in their late thirties or early forties.
- It can present with shorter duration of mass enlargement of around 5 years.
- It can be considered in the differential diagnoses of massive enlargement of breast.
- Given its rarity, accurately diagnosing a giant breast lipoma is crucial to prevent potential overtreatment, as it might otherwise be mistaken for a malignant tumour.

Lipomas are benign neoplasms composed of mature adipocytes and surrounded by a thin fibrous capsule^1^. They represent 16% of all mesenchymal tumours^2^. Twenty percent of all lipomas are located in the chest wall^3^. However, the incidence of breast lipomas is uncertain and is described as both common and uncommon in the literature^4^. Most lipomas are small and weigh only a few grams.

Giant lipomas are those measuring 10 cm or more in their largest diameter or weighing at least 1000 g^5^. Hawary *et al*.^6^. consider lipomas with diameters exceeding 5 cm and/or weighing 500 g as giant. Giant lipomas commonly occur in the thigh, upper back, and neck^5,7^. The breast is an uncommon site for giant lipomas. Accurately diagnosing a giant breast lipoma is essential to prevent unnecessary overtreatment, as it might otherwise be mistakenly treated as a malignant tumour due to its rarity.

Herein, we report a case of a woman with giant breast lipoma who underwent resection of the mass.

This case report has been reported in line with the SCARE Criteria 2023^8^.

## Case presentation

A 40-year-old woman from southern Nepal presented to the plastic surgery clinic with enlargement of the left breast for 5 years. The mass was gradually increasing in size over the years and was painless. She did not have nipple discharge or skin changes. She denied having a fever, loss of appetite, or weight. She did not give a history of any trauma to the region and denied taking hormonal medication or other drugs for concurrent medical conditions. No family members had a history of breast cancer or any other form of cancer within the first-degree relatives. She experienced menarche at the age of 14 and had regular menstrual cycles.

**Figure 1:**
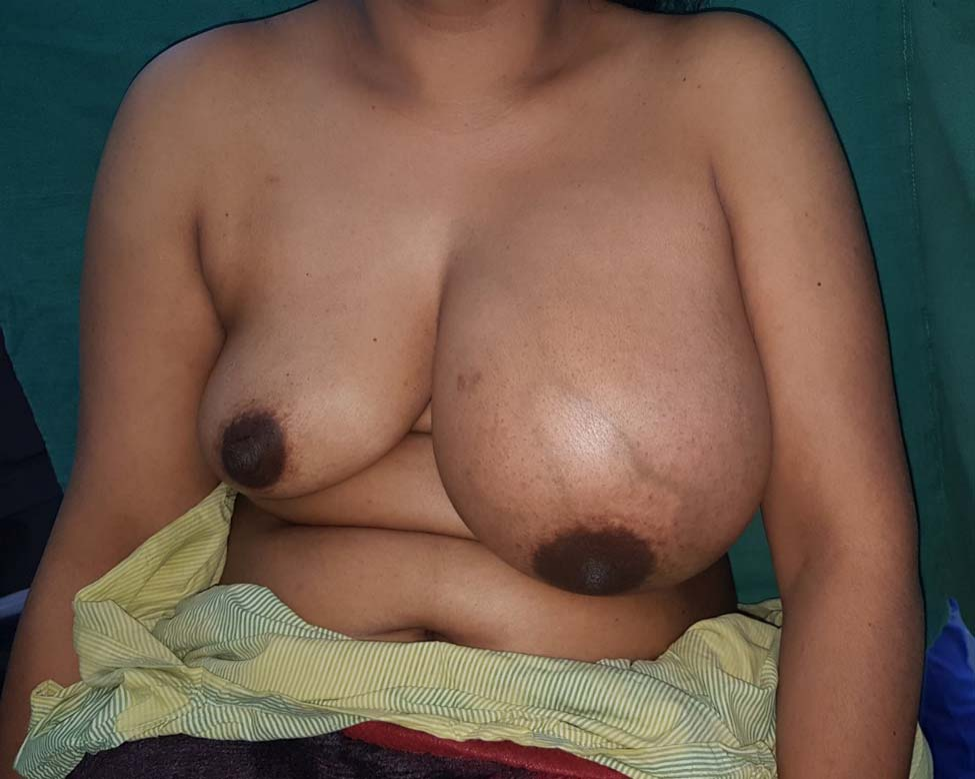
Picture of the patient with giant breast lipoma of the left breast before surgery.

A physical examination of the left breast revealed a well-defined, soft mass measuring 34 ×26×6 cm with dilated veins on the surface (Fig. 1). The overlying skin of the breast looked normal with a normal nipple and areola complex. No enlarged axillary lymph nodes were palpated. The right breast examination was unremarkable.

Ultrasonography (USG) of the breast showed a homogenously hyperechoic mass in the left chest wall deep to the left breast and pectoralis major muscle, without cystic area, calcifications, or internal vascularity. No axillary lymph node enlargement was detected. Mammography showed a heterogeneous lucent mass in the left retromammary region involving the pectoralis muscle with benign calcifications in the bilateral breast.

**Figure 2:**
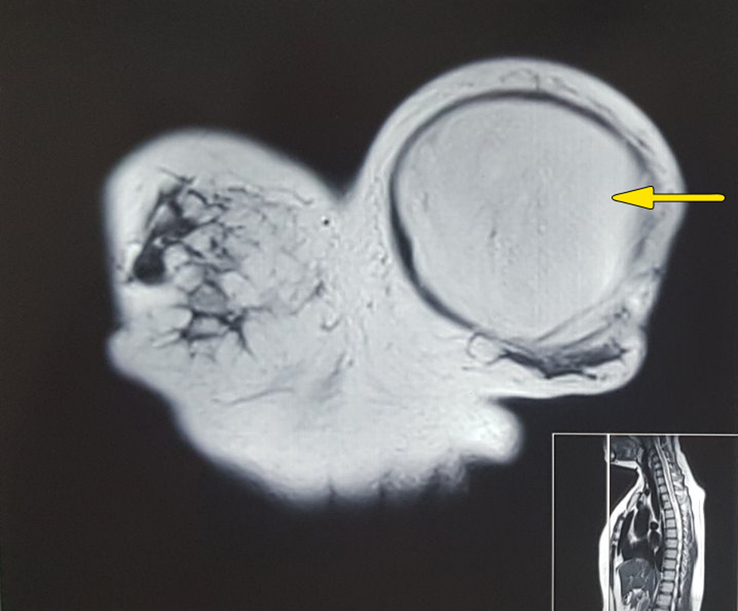
Magnetic resonance imaging showing well circumscribed homogeneous T2 high signal intensity lesion in the left breast (arrow); the lesion showed high signal intensity in T1 with suppression in STIR (not shown here).

Magnetic resonance imaging (MRI) of the chest demonstrated ~15×13×10.2 cm sized relatively well-defined homogenous T1/T2 high signal intensity mass showing signal suppression in fat-suppressed images in the left anterolateral chest wall (Fig. 2). Furthermore, the MRI revealed the mass to be encapsulated with multiple thin septa without thoracic extension or bony erosions. The pathological examination with core biopsy was not done, as history and physical examination suggested benign lesion which was supported by imaging. The patient elected to undergo surgery promptly to eliminate the disease at the earliest opportunity as she was concerned about cosmesis and the site of the disease.

**Figure 3:**
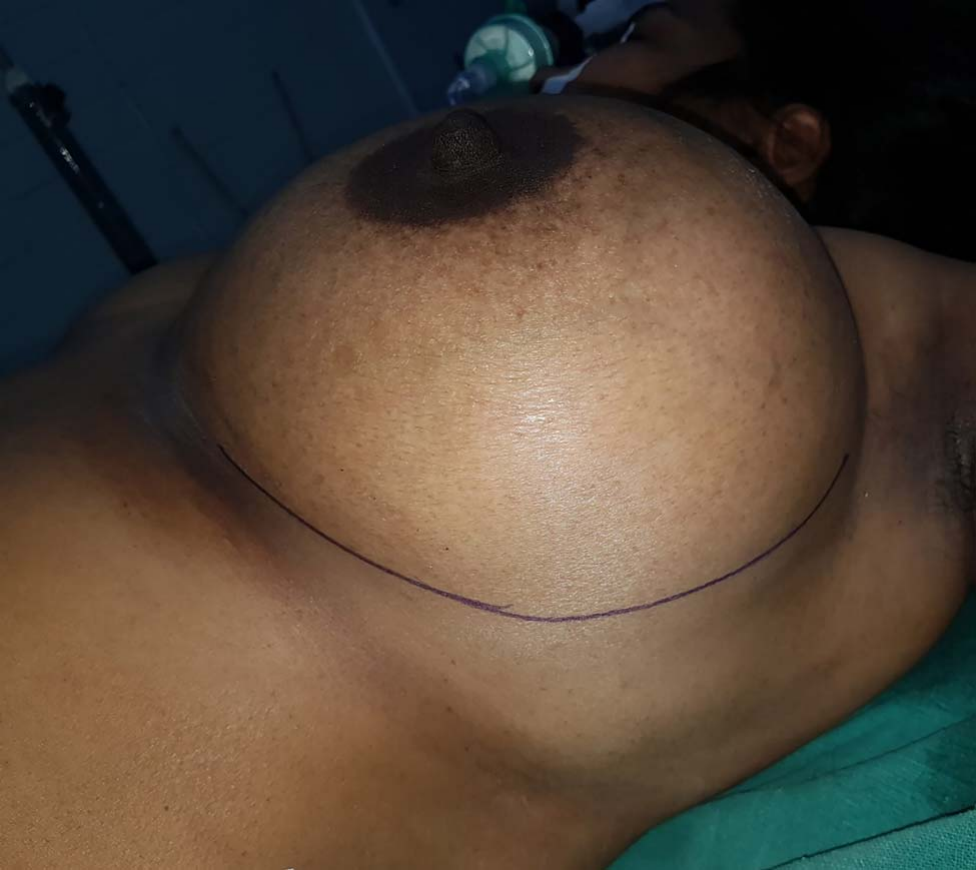
Preoperative marking over the site of incision.

**Figure 4:**
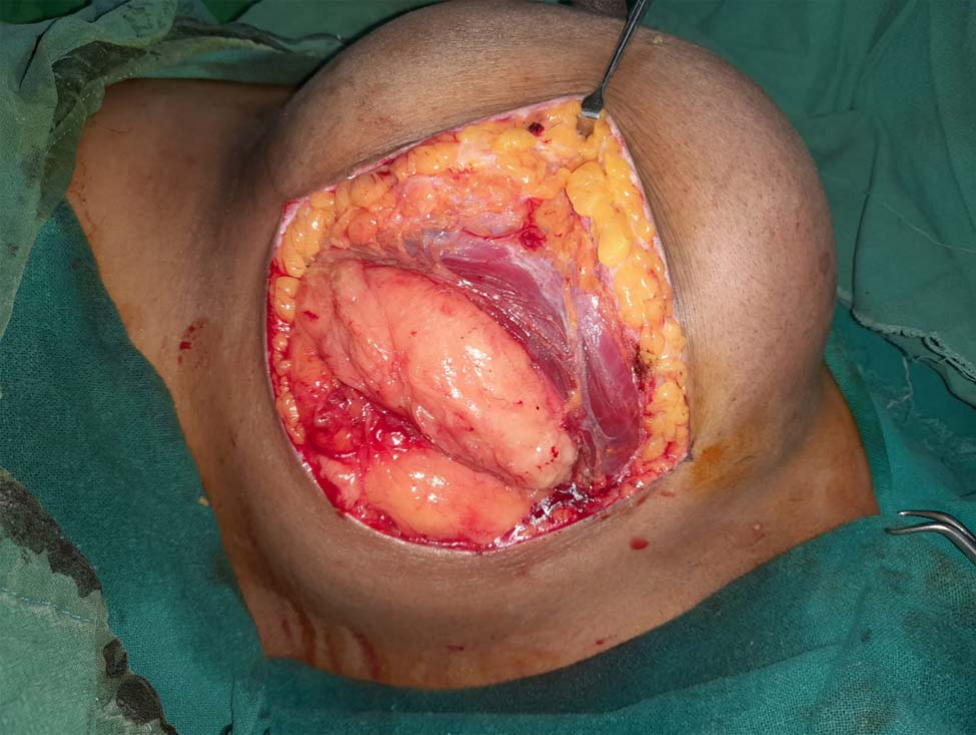
Subpectoral location of the giant breast lipoma.

**Figure 5:**
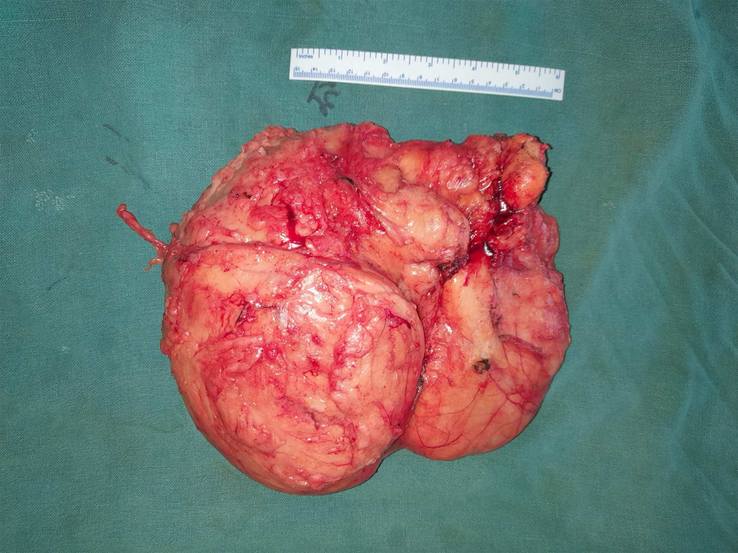
Excised specimen weighing 5 kg and measuring 20×18 cm.

**Figure 6:**
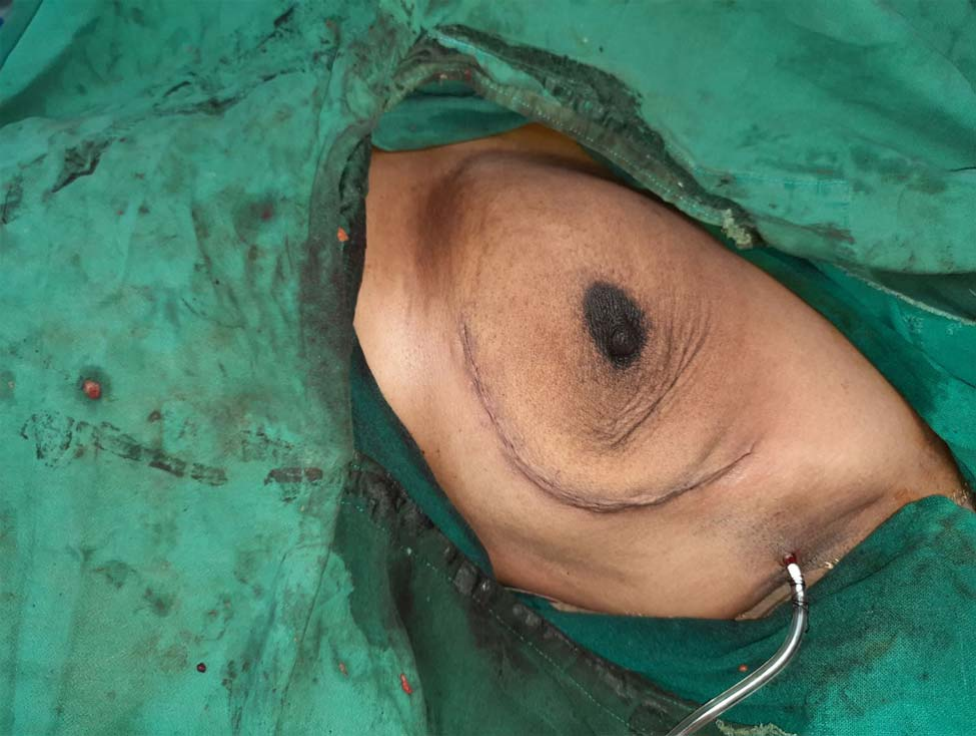
After the closure of the incision with a suction drain *in situ*.

The surgery was performed under general anaesthesia by the plastic surgery team. The mass was approached through a lateral curvilinear incision to preserve the inferior pedicle for possible need of a mastopexy procedure in the future (Fig. 3). Dermis and subcutaneous tissue were incised with cautery. Muscle was retracted after incision and eventually dissected. Vessels feeding the mass were ligated. The tumour was enucleated from underneath the pectoralis major muscle which was atrophic probably due to the compression effect of the mass (Fig. 4). However, pressure effect over the ribs was not noted. The entire mass weighed 5 kgs and measured 20×18 cm (Fig. 5). The incision was closed in layers over a closed suction tube drain and with cosmetic subcuticular sutures in the skin (Fig. 6). Pressure dressing was applied. The drain was removed on the 2nd postoperative day, and the postoperative period was uneventful.

**Figure 7:**
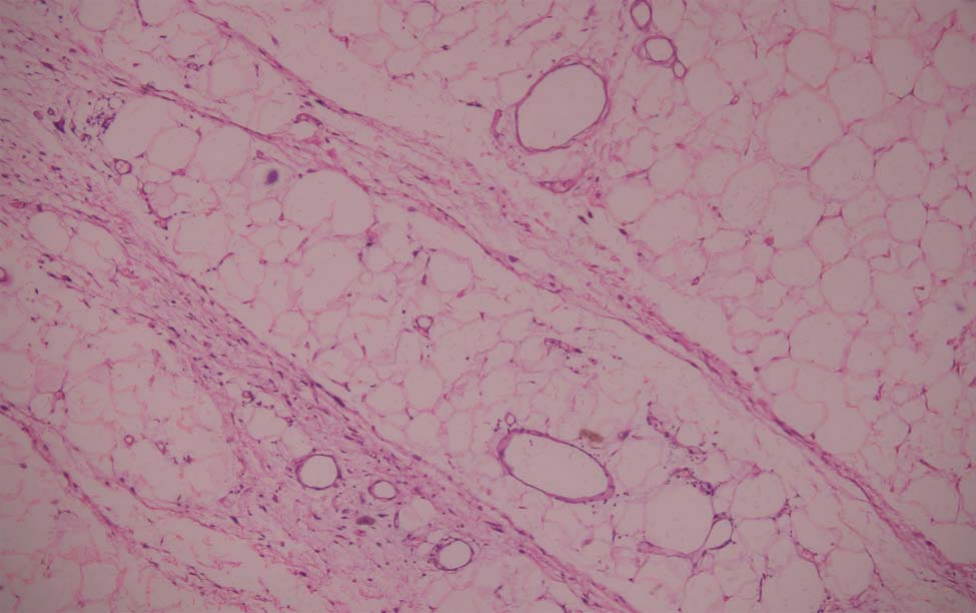
Histopathology of the specimen.

**Figure 8:**
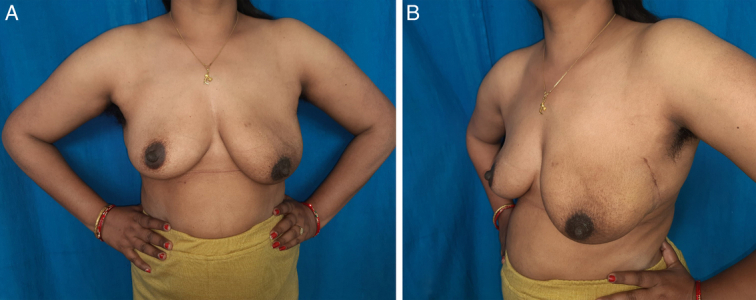
Two-month postoperative picture of the breast.

Histopathology of the surgical specimen revealed a well-encapsulated mass composed of lobules of mature adipocytes separated by fibrovascular septa without any atypical cells, mitotic figures, and necrosis features suggestive of lipoma (Fig. 7). After 2 months of follow-up in the outpatient clinic, the patient was satisfied with the outcome of the surgery (Fig. 8). She was advised for follow-up 6 monthly for the first year and yearly follow-up thereafter.

## Discussion

Lipoma was first described by Sir James Paget, in 1856, located within the trapezius muscle^9^. They are benign, slow-growing, primary mesenchymal tumours representing 4–5% of all benign tumours in the body^4^. The etiopathogenesis of lipomas is still uncertain; however, endocrine, inflammatory, mechanical mechanisms, and genetic defects may have an influence^10^.

Although some studies have mentioned breast lipomas to be common and some as uncommon, only a few cases of “giant” lipomas have been reported in the literature^4,11–17^. Rodriguez *et al.* ^18^. in 1997 reported the first case of giant breast lipoma weighing 1220 grams. The largest reported to date was that by Schmidt *et al.* ^19^. in a 64-year-old female measuring 50×40×30 cm and weighing 15.5 kg that developed over 25 years.

Breast lipomas tend to have a higher incidence in the 40–60 years age group, and most “giant” breast lipomas occur in the latter half of this group^4,19^. Yong *et al.* ^4^. and Nandkishor *et al.* ^15^. reported giant breast lipomas occurring in the early 50s and Schmidt *et al.* ^19^. in their early 60s; however, the patient in our study was comparatively younger of only 40 years of age when she presented to the clinic. Most case reports mention the duration of enlargement ranging from 10 years^15^ to 25–30 years^4,19^; however, in our case, it was only 5 years.

Lipomas are predominantly located in the subcutaneous tissue layer, nevertheless, intramuscular, subfascial, retroperitoneal, mediastinal, gastrointestinal, and intraneural localization has been discussed in the literature^20^. Most case reports of giant breast lipomas have not mentioned the location in terms of tissue layers (we assume it to be subdermal/subcutaneous, as these are more common locations in other body parts). The location in our case was underneath the pectoral major muscle.

Giant breast lipomas can mimic a wide variety of breast conditions. Giant masses in the breast can result from neoplastic conditions (fibroadenoma, phyllodes tumour, carcinoma, hamartoma, cyst) and non-neoplastic conditions (haematoma, abscess, galactoceles, steatonecrosis)^21^. Since our patient had longstanding history of unilateral breast enlargement, the major differentials to be ruled out included galactocele, steatonecrosis, phyllodes tumour, giant fibroadenoma, physiological breast hypertrophy and carcinoma. Distinguishing these conditions effectively solely through history and clinical examination is challenging, especially in the case of large and deep tumours where physical examination provides limited diagnostic assistance. Definitive identification can be achieved through simple radiography, ultrasonography, computed tomography, or magnetic resonance imaging^16^. Galactoceles and steatonecrosis do not have internal fibrous septa on imaging. Giant fibroadenoma and phyllodes tumour lack fat density on mammograms and appear as solid mass on USG. Mammography would reveal carcinoma as ill-defined mass and on sonography as irregular, hypoechoic mass with acoustic shadowing^21^. In our case, all the imaging modalities including mammography, USG and MRI have shown a lipoma-compatible lesion. Other differential diagnoses of giant lipomas are soft tissue tumours that can affect nerves, vessels, muscles, tendons, and even bones^22^.

Surgical excision and liposuction are two modalities of treatment of breast lipomas. Surgical excision is preferable to suction-assisted lipectomy (liposuction) because of large haematomas and recurrence due to incomplete removal of the tumour by liposuction^14^. Furthermore, liposuction can pose difficulty and have complications when the breast lipoma is submuscular as was in our case. It is also necessary to consider preserving the future pedicle of reduction mammoplasty/mastopexy in case there is a need to address excess skin and breast tissue following the excision of such large lesions. Also, complete removal of these lesions is important to avoid recurrences^4^ as many cases of recurrences have been reported and long-term follow-up may be necessary.

## Conclusion

A giant breast lipoma can be considered a differential diagnosis in a woman in her late thirties or early forties presenting with massive enlargement of the breast for about 5–30 years duration. Surgical excision of the same should be undertaken through a suitable approach keeping in mind the possibility of submuscular location and the future need to address the excess skin with a suitable mastopexy procedure.

## Ethical approval

According to Institutional Review Committee, ethical approval is not required for case reports.

## Consent

Written informed consent was obtained from the patient for publication of this case report and accompanying images. A copy of the written consent is available for review by the Editor-in-Chief of this journal on request.

## Sources of funding

This case report did not receive any specific grant from any funding agencies in the public, commercial or not-for-profit sectors.

## Author contribution

S.S. and A.R.: involved in the concept, collecting information, and manuscript writing. A.R. and A.C.: participated in the literature review and edited the draft. S.S. and J.M.S.: involved in the patient care team and also independently reviewed the manuscript. S.S., A.R., A.C., and J.M.S.: re-edited the draft and reshaped it into this manuscript. All authors approved the final version of the manuscript and agree to be accountable for all aspects of the work in ensuring that questions related to the accuracy or integrity of any part of the work are appropriately investigated and resolved.

## Conflicts of interest disclosure

The authors declare that there is no conflicts of interest regarding the publication of this paper.

## Research registration unique identifying number (UIN)

This study is case report and registration is not required.

1. Name of the registry: Not applicable.
2. Unique Identifying number or registration ID: Not applicable
3. Hyperlink to your specific registration (must be publicly accessible and will be checked): Not applicable.

## Guarantor

Samit Sharma, Aadesh Rayamajhi.

## Data availability statement

Not applicable.

## Provenance and peer review

Not commissioned, externally peer-reviewed.
